# Bleomycin Electrosclerotherapy and Skin Hyperpigmentation in Slow-flow Vascular Malformations: A Retrospective Monocentric Analysis

**DOI:** 10.1055/a-2783-4420

**Published:** 2026-02-06

**Authors:** Julius Henry Loeser, Dominik Schramm, Beatrix Rita Cucuruz, Alexander Gussew, Volker Stadie, Lilit Flöther, Olena Kaluheresku, Stefan Schob, Walter A Wohlgemuth

**Affiliations:** 1 University Hospital and Polyclinic for Radiology 14942 University Hospital HalleHalle (Saale)Sachsen-AnhaltGermany; 2 University Clinic and Polyclinic for Dermatology 14942 University Hospital HalleHalle (Saale)Sachsen-AnhaltGermany; 3 University Clinic for Anesthesiology and Operative Intensive Care Medicine 14942 University Hospital HalleHalle (Saale)Sachsen-AnhaltGermany

**Keywords:** Bleomycin electrosclerotherapy, Hyperpigmentation, Skin discoloration, Slow-flow vascular malformation

## Abstract

**Purpose:**

Sclerotherapy remains the standard for interventional treatment of slow-flow vascular malformations. However, bleomycin electrosclerotherapy (BEST) has shown promising results in the management of recurrent lesions. One notable adverse effect of BEST is the postinterventional development of skin hyperpigmentation. The aim of this study is the analysis of accidental skin hyperpigmentation after BEST of slow-flow vascular malformations.

**Materials and Methods:**

This retrospective study at our interdisciplinary vascular anomalies center investigated the occurrence of skin hyperpigmentation after bleomycin electrosclerotherapy of slow-flow vascular malformations over a period of 21 months documented clinically and in photographic findings, as well as related interventional treatment parameters with subsequent exploratory threshold analyses at 0.10 and 0.15 mg/kg. Subsequently, a comparison was made with recent publications reporting hyperpigmentation after bleomycin administration.

**Results:**

During the observation period, 72 patients were included with a total of 80 BEST procedures. Bleomycin-related skin hyperpigmentation was documented in 4 of 7 lymphatic malformation (LMs), 20 of 44 venous malformation (VM)and in 16 of 29 combined venolymphatic vascular malformations associated with other anomalies. On average, 27.1 application series of reversible electroporation per intervention were performed (range 1–85).

An average of 8.37 mg bleomycin was administered to LMs, 5.31 mg to VMs and 8.03 mg to the combined group in each session. Hyperpigmentation was more frequent with the hexagonal electrode: 33/56 (58,9 %); Needle-Foil Electrode (NFD) 6/19, Variable Geometry Device (VGD) 1/5. Rates were similar across entities (hexagonal: LM 3/5, VM 17/31, combined 13/20). Overall, bleomycin was administered intralesional 46 times with an average dose of 0.09 mg/kg bw (range 0.008–0.23) and intravenously 34 times, 0.22 mg/kg bw (range 0.13–0.5). Hyperpigmentation was more frequent after intravenous administration (61.8 %) than intralesional (41.3 %), likely reflecting higher dosing. A threshold dose of ≥0.10 mg/kg bleomycin was associated with a higher risk ratio for hyperpigmentation (RR 2.30, 95% CI 1.27–4.15).

**Conclusion:**

The frequency of skin hyperpigmentation following BEST seems to be analog to bleomycin-induced flagellate dermatitis and appears more frequently when using the hexagonal electrode and a higher bleomycin dosage per kg bodyweight (bw).

**Key Points:**

- Puncture related skin hyperpigmentation is a frequent but often underestimated adverse effect of Bleomycin electrosclerotherapy (BEST) in slow-flow vascular malformations.
- Our findings suggest that electrode geometry and puncture-related trauma contribute substantially to this side effect.
- Recognizing these factors allows for more informed electrode selection and patient counseling to minimize the risk and improve cosmetic outcomes after BEST.
- Exploratory analyses suggest higher mg/kg dosing increases hyperpigmentation risk. When clinically feasible, consider dose minimization especially in cosmetically sensitive areas.

**Citation Format:**

- Loeser JH, Schramm D, Cucuruz BR etal. Bleomycin Electrosclerotherapy and Skin Hyperpigmentation in Slow-flow Vascular Malformations: A Retrospective Monocentric Analysis. Rofo 2026; DOI 10.1055/a-2783-4420

## Zusammenfassung

**Ziel:**

Analyse von Häufigkeit und Einflussfaktoren von Verfärbungen der Haut nach Bleomycinelektrosklerotherapie (BEST) bei slow-flow-Gefäßmalformationen.

**Materialien und Methoden:**

Monozentrische, retrospektive Registeranalyse über 21 Monate. Es wurden klinische und fotografische Befunde erfasst sowie Elektrodenarten, Applikationswege und Dosen (mg/kg KG) mit nachfolgenden explorativen Schwellenanalysen bei 0,10 und 0,15 mg/kg. Im Anschluss daran wurde ein Vergleich mit aktuellen Veröffentlichungen durchgeführt, in denen über Hyperpigmentierung nach Bleomycin-Verabreichung berichtet wurde.

**Ergebnisse:**

72 Patienten mit insgesamt 80 BEST-Prozeduren. Bleomycin-induzierte Hautverfärbungen traten in 40/80 (50%) Interventionen auf, darunter lymphatische Malformationen (LM) 4/7, venöse Malformationen (VM) 20/44, kombiniert 16/29 mit vergleichbaren Raten zwischen den Entitäten, jedoch Unterschieden zwischen den Elektrodenarten: hexagonaler Elektrode: 33/56 (58,9%), Needle-Foil Electrode (NFD) 6/19, Variable Geometry Device (VGD) 1/5. Bei intravenöser Gabe des Bleomycin waren Hyperpigmentierung häufiger als nach intraläsionaler Gabe (21/34 [61,8%] vs 19/46 [41,3%]). Explorativ zeigte sich bei höherer Dosis mit einem Schwellenwert von ≥ 0,10 mg/kg als auch ≥0,15 mg/kg ein erhöhtes relatives Risiko für Diskolorationen der Haut (RR 2,30; 95%-KI 1,27–4,15).

**Schlussfolgerung:**

Diskolorationen der Haut nach BEST bei slow-flow-Gefäßmalformationen ähneln der Bleomycin-induzierten Flagellantendermatitis. Das Risiko scheint v.a. mit der hexagonalen Elektrode und höherer mg/kg-Dosis erhöht zu sein; zwischen den Entitäten zeigen sich vergleichbare Raten.

**Kernaussagen:**

- Punktionsbedingte Hauthyperpigmentierung ist eine häufige, aber oft unterschätzte Nebenwirkung der Bleomycinelektrosklerotherapie (BEST) bei slow-flow Gefäßmalformationen.
- Unsere Ergebnisse deuten darauf hin, dass die Geometrie der Elektrode und das durch die Punktion verursachte Trauma eine wichtige Rolle bei dieser Nebenwirkung spielen.
- Die Kenntnis dieser Faktoren ermöglicht eine fundiertere Auswahl der Elektrode und eine bessere Beratung der Patienten, um das Risiko zu minimieren und die kosmetischen Ergebnisse nach der BEST zu verbessern.
- Explorative Analysen deuten darauf hin, dass eine höhere Dosierung in mg/kg das Risiko einer Hyperpigmentierung erhöht. Wenn klinisch möglich, sollte eine Dosisminimierung in Betracht gezogen werden, insbesondere in kosmetisch sensiblen Bereichen.

## Introduction

According to the current ISSVA classification, vascular malformations can be divided into fast-flow malformations, including arteriovenous malformations, and slow-flow malformations, including capillary (CM), lymphatic (LM), and venous malformations (VM) as well as several syndromes 1 2 . Although congenital, these malformations may become symptomatic or clinically apparent at various ages. Typical symptoms are pain, swelling, functional limitations, and recurrent infections of the affected area as well as recurrent thrombophlebitis and thrombosis 3 . Over the last few years, the effectiveness of bleomycin electrosclerotherapy (BEST) on slow-flow vascular malformations has been demonstrated in several studies 4 5 6 7 8 . Bleomycin, known since the 1960s, is still used today in various chemotherapy protocols as well as in electrochemotherapy, which combines bleomycin administration with subsequent short, reversible electroporation pulses for the treatment of various skin tumors 9 . The generation of short electrical pulses between individual needle electrodes (reversible electroporation) leads to a short-term change in the structure of the cell membrane, causing it to become temporarily more permeable and thereby increasing the intracellular concentration of molecules like bleomycin, that normally penetrate the cell membrane only in very small quantities. BEST utilizes these effects for the treatment of slow-flow vascular malformations by using lower doses of bleomycin as compared to conventional bleomycin sclerotherapy and a broad selection of needle electrodes.

Serious bleomycin-induced side effects (e.g. bleomycin-induced pulmonary fibrosis) in the context of conventional bleomycin sclerotherapy seem to be very rare due to the low applied doses and have only been described in a few cases 8 10 11 .

However, a more frequently observed adverse event reported in studies to this day are local skin discolorations following conventional sclerotherapy with bleomycin and BEST, which normally fade over time but can sometimes still be visible years after treatment 5 10 12 13 14 . Other localized dermal lesions in the form of necrosis, secondary infections, etc. have also been described, but seem to be rare 10 .

When bleomycin is used, for example, in the chemotherapy of Hodgkinʼs lymphoma, local, brownish skin discoloration has also been repeatedly described, and is known as bleomycin-induced flagellate hyperpigmentation 15 16 17 18 . The term “flagellate” derives from Latin and refers to the whip-like pattern of skin discoloration, typically triggered by scratching or minor skin trauma. Typically, this occurs after the application of high doses of bleomycin (100–300 mg); however, discoloration has also been described at much lower levels 17 19 .

Several hypotheses have been proposed, but the precise underlying mechanisms remain under investigation. For example, increased melanin levels due to various intercellular interactions, toxic effects of bleomycin itself, and induction through mechanical interactions, e.g. through scratching of clothing, are hypothesized 19 20 21 22 . The aim of this retrospective study was to determine the frequency and potential risk factors associated with bleomycin-induced skin discoloration following BEST.

## Materials and Methods

### Study design

The study consisted of a retrospective analysis based on a local prospective, consecutive registry at our tertiary care Interdisciplinary Vascular Anomalies Center. This analysis included all patients with slow-flow malformations who were treated between January 2021 and January 2024. The study was approved by the local ethics committee. Written informed consent was obtained from the patients or their legal guardians if they were still underage.

Over a period of 21 months, 72 out of 121 patients with a slow-flow vascular malformation were included in this monocentric and retrospective study based on the following inclusion and exclusion criteria. For inclusion, patients had to be treated with a BEST of their slow-flow vascular malformation, symptoms before and after the intervention had to be documented both immediately postoperatively and during the course of the intervention until the follow-up no earlier than three months after the procedure. Furthermore, only patients who received photo documentation of the treated area before the intervention and during follow-up were included. Patients were excluded from the study for the following reasons: they had previously received a cumulative bleomycin dose of more than 100 mg; they were pregnant or breastfeeding; they had previously received radiation to the treatment areas; they had a malformation component that could not be classified as a slow-flow malformation, or they had a known chronic pain syndrome.

The diagnosis and ISSVA-based subclassification of a slow-flow malformation were established in an interdisciplinary vascular anomalies conference at our VAC, integrating the documented patient’s history, clinical examination, ultrasound (US), MRI and, where available, histology. As no universally accepted quantitative flow cut-off exists, this classification inevitably retained a partially subjective component.

Skin discolorations were analyzed on a per-procedure basis.

BEST was performed in accordance with the current operating procedure 13 . Thus, the malformation was punctured with ultrasound guidance and contrast was injected under fluoroscopy. Bleomycin was administered either intralesionally or intravenously, depending on the malformation’s extent, location, and presence of communicating or draining components. The administered dose of bleomycin depended in particular on the form of administration (iv or intralesional) and the overall extent of the malformation. Following bleomycin injection, the targeted area of the malformation was punctured with needle electrodes to apply electrical pulses. Different needle electrode configurations were available to suit the specific location and extent of the malformation: finger electrodes of the needle-foil design (NFD), Variable Geometry Device design (VGD) series as freely positionable needles and hexagonal configuration electrodes. There are seven parallel needles incorporated in a fixed hexagonal configuration in the hexagonal electrodes, six parallel needles in a fixed rectangular configuration in the NFD series, whereas the VGD needle electrodes consist of individual longer needles that may be freely arranged (e.g. three needles in a triangle) in parallel to perform electroporation. Electroporation protocols, differing mainly in electric field strength, were automatically generated by the device: 750 V/cm for hexagonal electrodes, 600 V/cm for NFD series, and 1,000 V/cm for VGD series.

Once reversible electroporation was completed, the treated sites were bandaged and the further postoperative course documented until the patient could be discharged. Our center’s interdisciplinary team also supported patients after hospital discharge, and any adverse events and complications were documented and photographed. During standard follow-up three months after the intervention to ensure the patientʼs subsequent care, the symptoms, such as pain, swelling, and functional limitations, were documented once again and photographs of the area treated were taken. Postoperative complications were additionally categorized using the Clavien-Dindo classification 23 . Changes in symptoms before the intervention and at the earliest three months afterwards were categorized as aggravated, unchanged, improved, and asymptomatic.

Bleomycin dose was analyzed as mg/kg body weight. Exploratory threshold analyses (0.10 and 0.15 mg/kg) were conducted based on the cohort’s dosing distribution. Risk ratios (RRs) with 95% confidence intervals and two-sided Fisher’s exact test p-values were calculated.

Continuous variables were assessed for normality using the Shapiro-Wilk test. Non-normally distributed data are summarized as median and range (median (range)), whereas approximately normally distributed data are presented as mean and standard deviation (mean (SD)). Categorical variables are reported as absolute and relative frequencies, n (%).

## Results

Out of the 72 (100%) patients included in this study, 26/72 (36.1%) were male and 46/72 (63.9%) females. The median age at treatment was 14.0 years (range 2–61). In terms of malformation types (on a patient-level), seven lymphatic malformations (LM), 37 venous malformations (VM), and 28 combined venolymphatic or complex malformations associated with other anomalies were treated, amounting to 80 treated lesions in 72 patients. Eight patients underwent treatment for two separate malformations parts. Follow-up was performed after a median of 11 months (range 3–26).

The main symptoms before BEST were pain (n = 60), swelling (n = 32), lymphorrhea (n = 7), recurrent thrombophlebitis (n = 22), and recurrent infections including erysipelas (n = 8). According to the Clavien-Dindo classification, four postoperative wound infections that required antibiotics and one skin ulceration, which healed over a period of about three months were observed (Grade II) while no other complications occurred.

The median number of reversible electroporation series per BEST session was 23 (range 1–85) including a 23 series for LMs (range 5–40), 24.6 series for VMs (range 1–69) and 31.86 series for combined malformation components (range 3–85).

Using the hexagonal electrode (per procedure level), electroporation was performed in 29.7 series per intervention (range 3–69), with the NFD finger electrodes 19.79 (range 5–44) and VGD electrodes 21.8 (range 1–85).

In total, the hexagonal electrodes were used 56 times, including five times for LMs, 33 times for VMs and 18 times for combined venolymphatic malformation components or vascular malformations associated with other anomalies. Among these, skin discoloration was observed in 33/56 cases (58.9%) distributed as three in LM, 17 in VM, and 13 in combined malformations. The electrodes of the NFD series were used in 19 procedures (26%), with one discoloration in LM, two in VM and three in combined malformation components, corresponding to 6/19 (31.6%) of NFD procedures. Five times VGD-type electrodes were used, with only one discoloration in a VM (1/5; 20%).

**Fig. 1:**
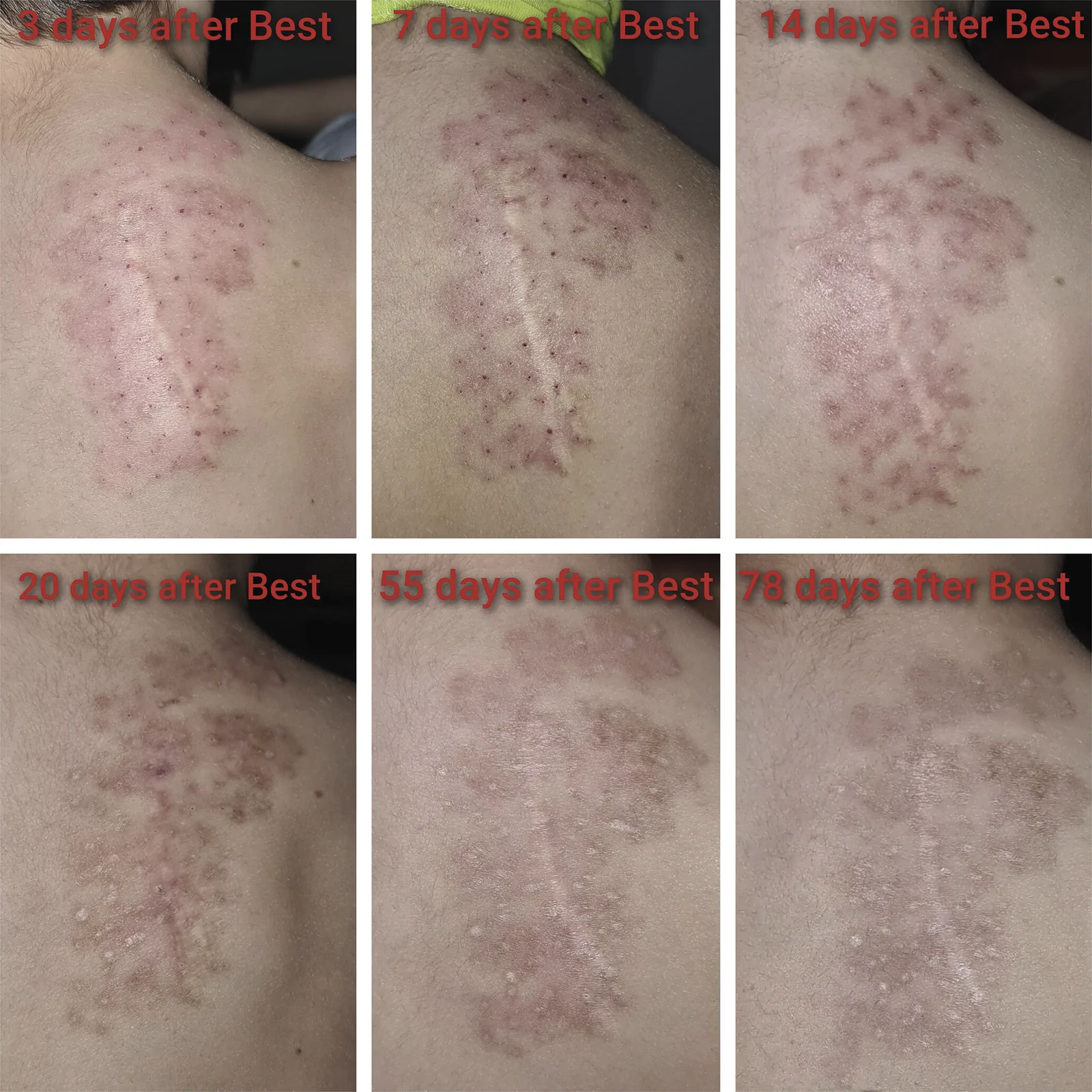
Development of the skin reaction over time after BEST in a venous malformation on the back, shown as a collage arranged from left to right and top to bottom, demonstrating gradual fading of the puncture site discoloration. The coloration changes from a reddish hue to a more brownish color. A scar from a previous operation done some time before BEST can also be seen.

**Fig. 2:**
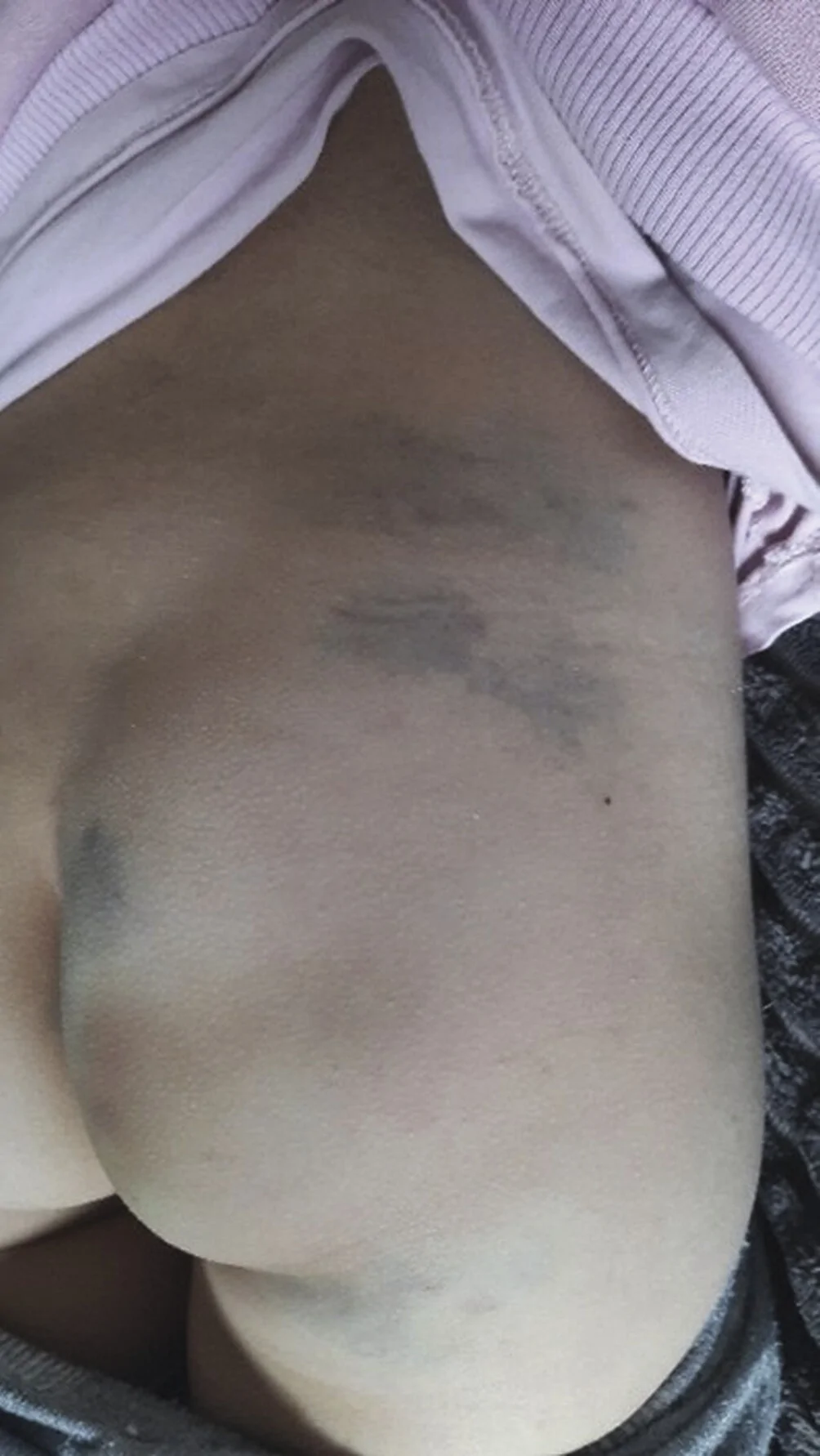
Images of a patient with a known gluteal venous malformation. On the left is directly before BEST, in the middle 12 months after BEST, and on the right 26 months after BEST. It is noticeable that the brownish discoloration in the area of the malformation treated with the hexagonal electrode fades markedly.

**Fig. 3:**
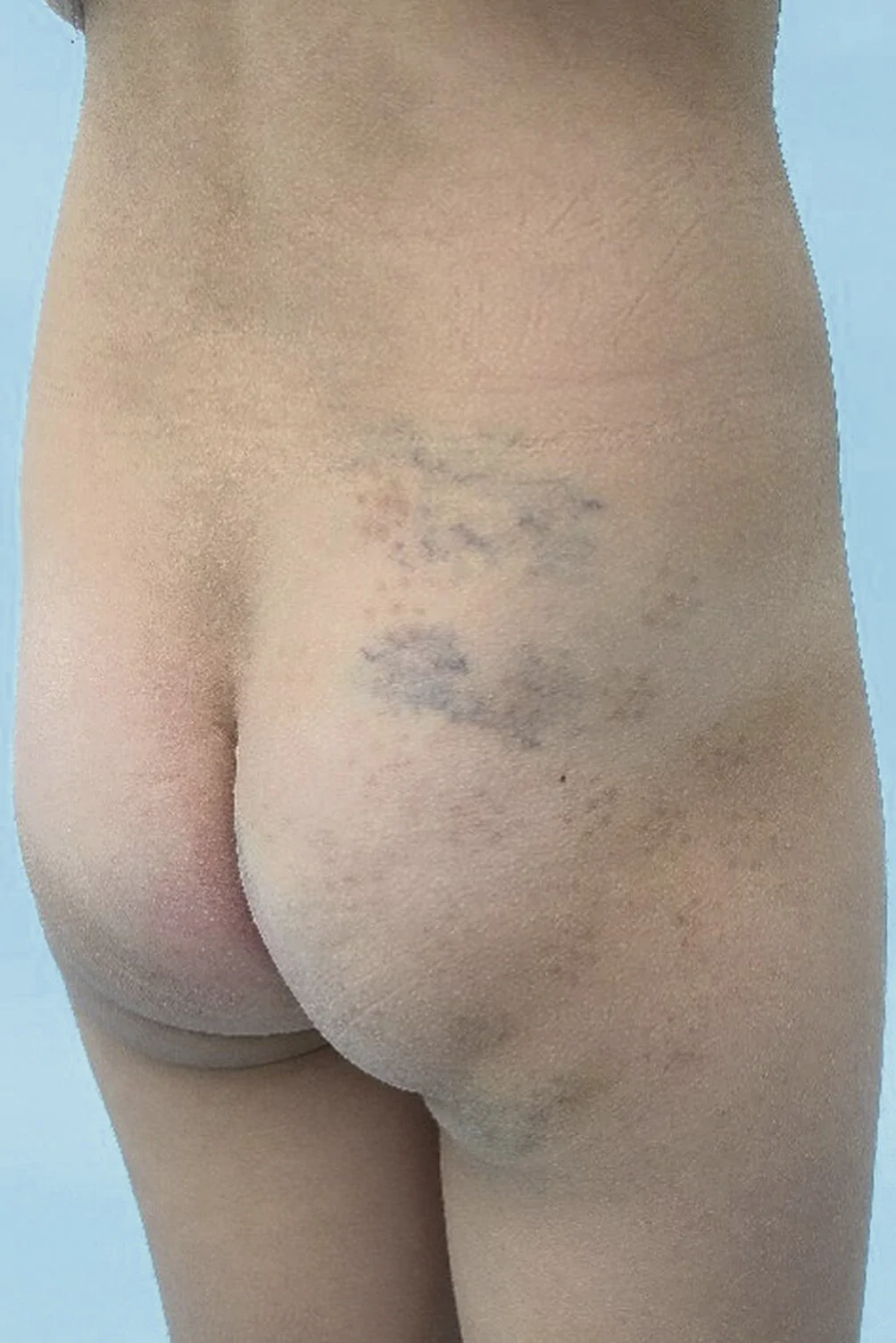
Images of a patient with a known gluteal venous malformation. On the left is directly before BEST, in the middle 12 months after BEST, and on the right 26 months after BEST. It is noticeable that the brownish discoloration in the area of the malformation treated with the hexagonal electrode fades markedly.

**Fig. 4:**
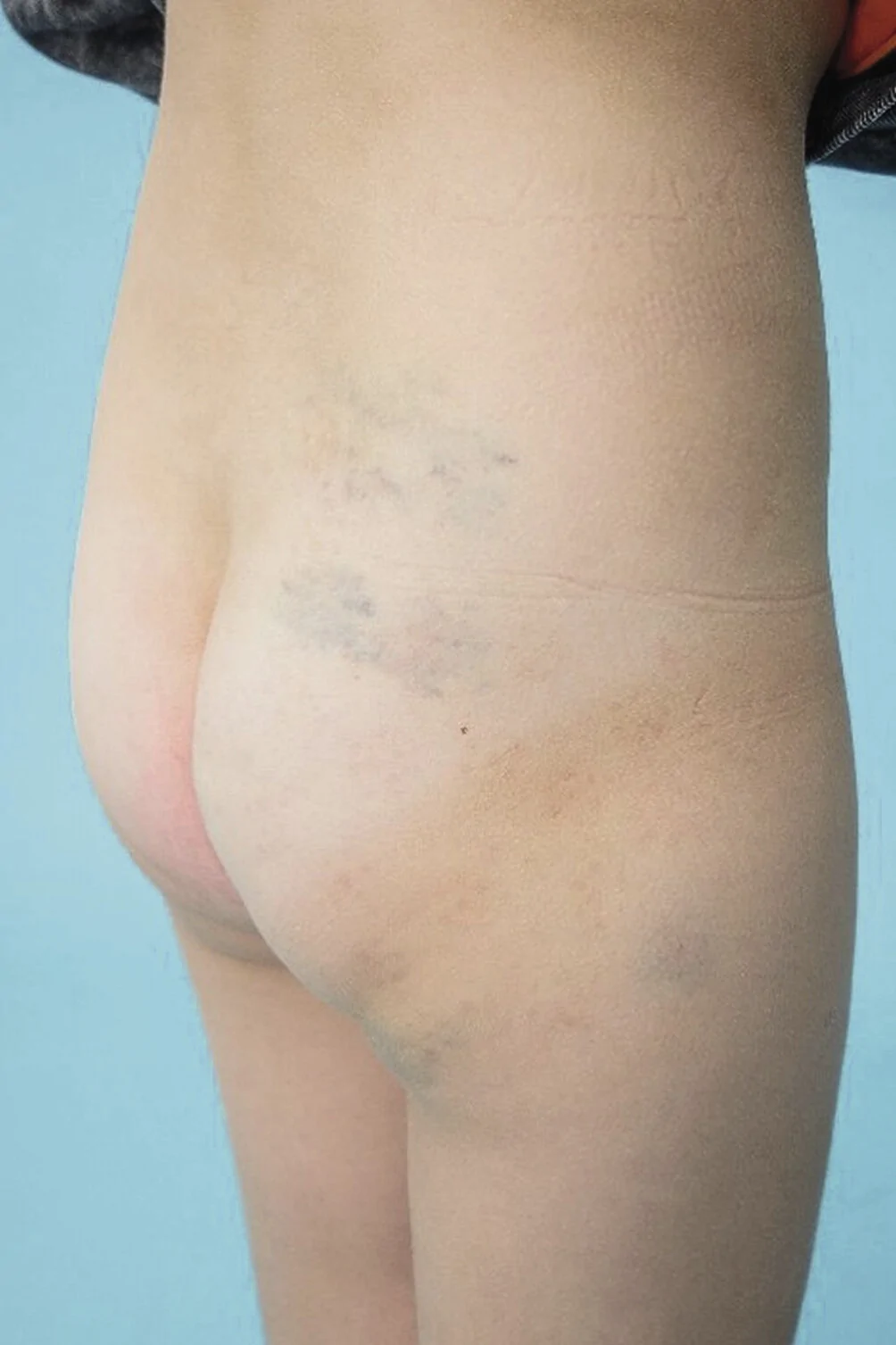
Images of a patient with a known gluteal venous malformation. On the left is directly before BEST, in the middle 12 months after BEST, and on the right 26 months after BEST. It is noticeable that the brownish discoloration in the area of the malformation treated with the hexagonal electrode fades markedly.

Of the total of 38/72 (52.8%) patients with documented discoloration during follow-up, 23/72 (31.9%; 23/38, 60.5%) responded for a further evaluation at the time of data collection. Of these 23, 20 patients reported a fading of the discoloration, whereby in 13 cases the discoloration could only be clearly identified by the experienced eye and with the knowledge of the existing documentation but it was no longer noticed by the patients themselves ( Fig. 1 , Fig. 2 , Fig. 3 , Fig. 4 ). Progression was not recorded in any case. The mean follow-up interval among the 23 respondents was 19.2 months (SD 5.2; range 8–28). A median bleomycin dose of 4.0 mg (range 0.75–18.0) was applied per procedure, including 8.37 mg for LM (range 0.8–15), 5.31 mg for VM (range 0.75–15) and 8.03 mg for combined malformation components (range 1.5–18). The median cumulative bleomycin dose per patient in the study period was 4.0 mg (range 0.8–19). The bleomycin dose for the hexagonal electrode was 7.85 mg (range 0.75–18), for NFD electrodes, 3.02 mg (range 0.8–15), and for VGD electrodes, 5.7 mg (range 1–17). Across all procedures, the median bleomycin dose was 0.14 mg/kg (range 0.008–0.50 mg/kg). A dose of 0.09 mg/kg bw was used for intralesional administration (range 0.008–0.23) with 46 sessions, and 0.22 mg/kg bw for intravenous administration (range 0.13–0.50) with 34 sessions.

A total of 19 discolorations (19/46; 41.3%) occurred after intralesional administration of bleomycin and a total of 21 after intravenous administration (21/34; 61.8%), thus there was only a trend but not a significant difference.

In exploratory analyses, skin discoloration was more frequent at ≥ 0.10 mg/kg than < 0.10 mg/kg (31/48 [64.6%] vs 9/32 [28.1%]; RR 2.30, 95% CI 1.27–4.15; p = 0.0027). Using a 0.15 mg/kg threshold, a consistent path (25/38 vs 15/42; RR 1.84, 95% CI 1.16–2.94; p = 0.013) was observed. Within hexagonal electrodes, the association persisted at ≥ 0.10 mg/kg (RR 2.65; p = 0.0016) and remained present at 0.15 mg/kg (23/32 vs 10/24; RR 1.73; p = 0.030).

After BEST, symptoms progressed in only one case (1/80; 1.3%) (with increased pain and persistent thrombophlebitis), remained unchanged in nine cases (9/80; 11.3%) (pain n = 5, swelling n = 2, erysipelas n = 1, lymphorrhea n = 1), and resolved completely in three previously symptomatic cases (3/80; 3.8%), in whom pain (n = 2), thrombophlebitis (n = 1), erysipelas (n = 1), and lymphorrhea (n = 1) disappeared. In 67 (67/80; 83.8%) all documented symptoms improved.

## Discussion

BEST was introduced several years ago as a new procedure for the treatment of slow-flow vascular malformations that is being evaluated in an increasing number of studies. Several scientific studies have demonstrated its feasibility, successful therapeutic outcome and relatively minor adverse events 4 5 6 7 24 25 . After 80 BESTs, a considerable improvement in symptoms was also seen in most of our patients in this study at follow-up.

However, brownish skin discolorations in the area where the needle electrodes were inserted was the most common side effect ( Fig. 1 , Fig. 2 , Fig. 3 , Fig. 4 ). The aim of this retrospective, monocentric study was to analyze the occurrence and underlying risk factors of skin discolorations more closely.

**Fig. 5:**
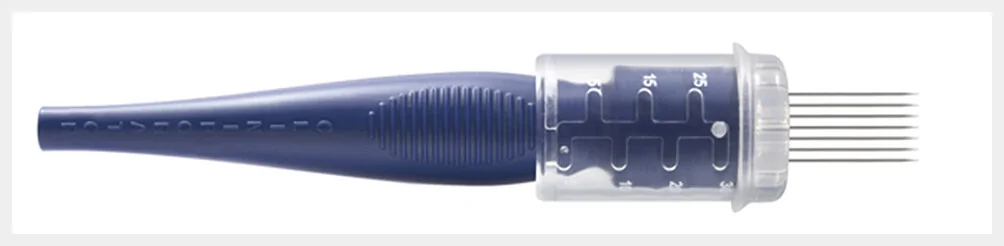
Needle electrodes used for BEST (hexagonal, NFD-Series, VGD-Series).

**Fig. 6:**
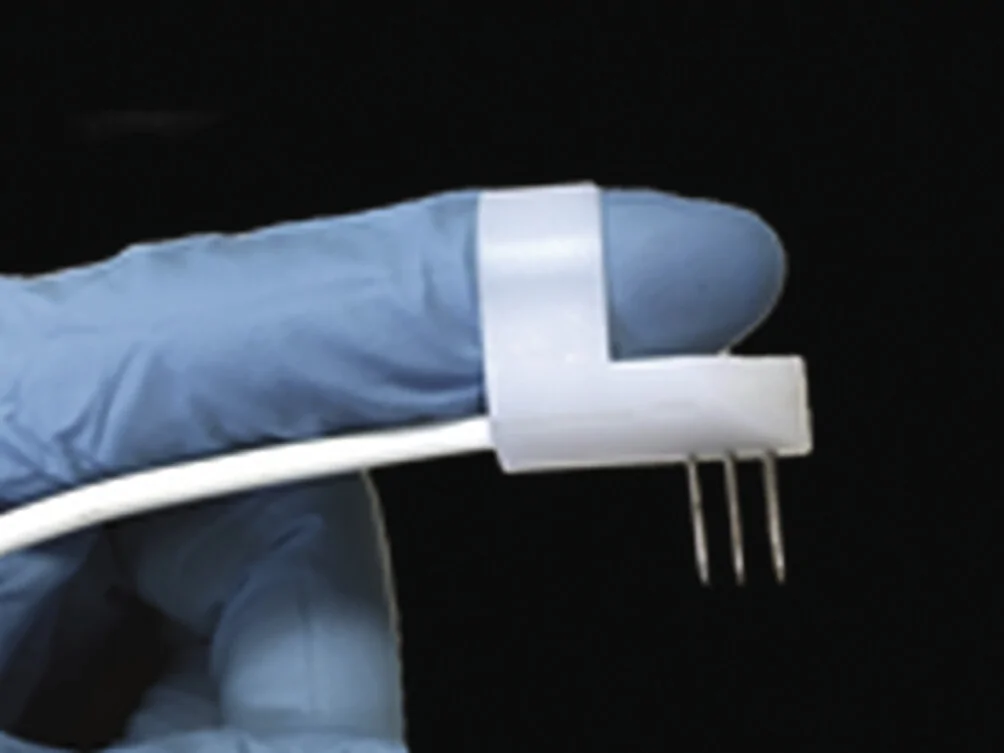
Needle electrodes used for BEST (hexagonal, NFD-Series, VGD-Series).

**Fig. 7:**
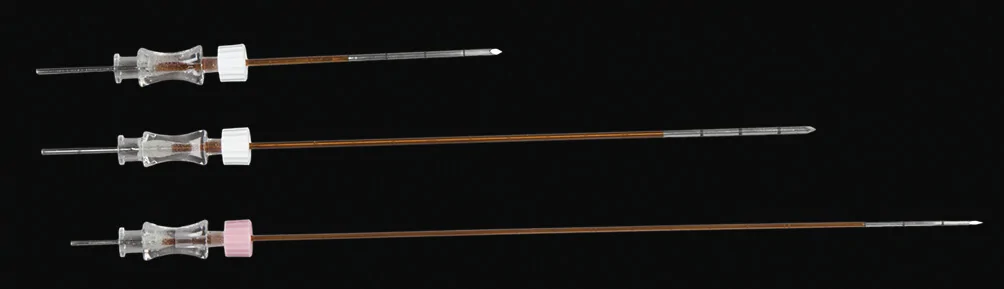
Needle electrodes used for BEST (hexagonal, NFD-Series, VGD-Series).

In our cohort, the vast majority of discolorations occurred in procedures performed with hexagonal electrodes, whereas NFD and VGD electrodes accounted for only a small minority of events ( Fig. 5 , Fig. 6 , Fig. 7 ). This suggests that the bleomycin dose alone is not the major risk factor, but the geometry of the electrodes in accordance with the applied electrical pulses plus the number of skin punctures by the needles may play an additional and substantial role in development of this side effect.

Procedures performed with hexagonal electrodes were also associated with higher bleomycin doses than those using NFD or VGD electrodes, and exploratory dose-threshold analyses indicated a higher proportion of discolorations at doses above 0.10 and 0.15 mg/kg. Together, these findings support a dose-response relationship with a potential additional contribution of electrode geometry and puncture-related trauma. However, the study’s retrospective design and lack of pre-specified thresholds mean that these results should be interpreted as hypothesis-generating.

Overall, our findings are consistent with recently published BEST series, in which cutaneous discoloration is described as a common but usually transient adverse effect in pediatric and lymphatic malformation cohorts 26 27 28 .

Nevertheless, skin discoloration was also observed with NFD and VGD electrodes despite lower average doses. On the other hand, studies and case reports have sporadically described the same phenomenon, suggesting the hypothesis that high bleomycin doses may tend to lead to more skin discoloration, but not exclusively, and that discoloration is more likely to be caused by other factors 13 14 17 18 19 .

Based on the results, the applied electrical field strength does not seem to play a crucial role, as documented by the comparatively high field strength of the VGD series electrodes and the comparatively low number of skin discolorations.

In contrast to the skin discolorations at the puncture sites described here, a striped to streak-like aspect characterizes bleomycin-induced flagellate dermatitis, well described within published studies, although the color is quite similar with the application of bleomycin being the connecting element in both conditions 18 29 . In BEST, the bleomycin dose is considerably lower compared to the other possible uses in oncology, electrochemotherapy, and in conventional Bleomycin sclerotherapy. It is applied either intralesional or intravenously 30 .

In conclusion, even if the specific pathomechanism is still unknown, a local accumulation of bleomycin induced by the puncture-related minor skin trauma appears reasonable 20 22 . This would also explain the frequent occurrence of discolorations when using the hexagonal electrode, as it requires with its seven hexagonally arranged needles a stronger force for the insertion than the NFD and VGD electrodes and an increased risk of potential redistribution of bleomycin from the treated tissue to the skin via multiple puncture tracks.

Furthermore, there is the fact that the needles may become blunter with repetitive applications as the number of electroporation series increases, and therefore the trauma to the skin may further intensify.

Furthermore, observations from the early days of BEST with similar discolorations aside from the treated area, on which adhesive bandages, for example, from ECG electrodes, were quickly removed in a traumatizing fashion, match these findings. In line with the existing literature, most discolorations in our cohort gradually faded over time, and only a minority of patients continued to notice residual changes at follow-up 12 .

The monocentric and particularly retrospective design of the study is its greatest limitation. Further prospective studies are needed to investigate the interactions between the skin and bleomycin, especially in the context of BEST. Until further data is published, careful handling of the patientʼs skin seems reasonable to avoid microtrauma during BEST and certainly to prevent skin discolorations 13 . This can also be considered when choosing the electrode, although the decision should primarily be based on the localization, type, and extent of the malformation.

## Conclusion

Puncture-related skin discolorations after BEST appear to be analogous to bleomycin-induced flagellate dermatitis with an increased tendency to appear when using hexagonal electrodes; exploratory dose analyses further suggest an increased risk at higher mg/kg dosing.

## Notes

Clinical relevance of the study

1. Puncture related skin discoloration is a frequent but often underestimated adverse effect of bleomycin electrosclerotherapy (BEST) in slow-flow vascular malformations.
2. Our findings suggest that electrode geometry and puncture-related trauma contribute substantially to this side effect.
3. Recognizing these factors allows for more informed electrode selection and patient counseling to minimize the risk and improve cosmetic outcomes after BEST.
4. Exploratory analyses suggest higher mg/kg dosing increases skin discoloration risk. When clinically feasible, consider dose minimization, especially in cosmetically sensitive areas.
